# Long-term cryopreservation of decellularised oesophagi for tissue engineering clinical application

**DOI:** 10.1371/journal.pone.0179341

**Published:** 2017-06-09

**Authors:** Luca Urbani, Panagiotis Maghsoudlou, Anna Milan, Maria Menikou, Charlotte Klara Hagen, Giorgia Totonelli, Carlotta Camilli, Simon Eaton, Alan Burns, Alessandro Olivo, Paolo De Coppi

**Affiliations:** 1 Great Ormond Street Institute of Child Health, UCL, London, United Kingdom; 2 Department of Medical Physics and Biomedical Engineering, UCL, London, United Kingdom; 3 Department of Clinical Genetics, Erasmus Medical Centre, Rotterdam, The Netherlands; Michigan Technological University, UNITED STATES

## Abstract

Oesophageal tissue engineering is a therapeutic alternative when oesophageal replacement is required. Decellularised scaffolds are ideal as they are derived from tissue-specific extracellular matrix and are non-immunogenic. However, appropriate preservation may significantly affect scaffold behaviour. Here we aim to prove that an effective method for short- and long-term preservation can be applied to tissue engineered products allowing their translation to clinical application. Rabbit oesophagi were decellularised using the detergent-enzymatic treatment (DET), a combination of deionised water, sodium deoxycholate and DNase-I. Samples were stored in phosphate-buffered saline solution at 4°C (**4°C**) or slow cooled in medium with 10% Me2SO at -1°C/min followed by storage in liquid nitrogen (**SCM**). Structural and functional analyses were performed prior to and after 2 and 4 weeks and 3 and 6 months of storage under each condition. Efficient decellularisation was achieved after 2 cycles of DET as determined with histology and DNA quantification, with preservation of the ECM. Only the SCM method, commonly used for cell storage, maintained the architecture and biomechanical properties of the scaffold up to 6 months. On the contrary, 4°C method was effective for short-term storage but led to a progressive distortion and degradation of the tissue architecture at the following time points. Efficient storage allows a timely use of decellularised oesophagi, essential for clinical translation. Here we describe that slow cooling with cryoprotectant solution in liquid nitrogen vapour leads to reliable long-term storage of decellularised oesophageal scaffolds for tissue engineering purposes.

## Introduction

Tissue engineering can offer effective alternatives to conventional treatments through the development of bio-constructs that can restore or replace damaged tissue and organs. To fully respond to rising clinical needs it is now necessary to reconsider tissue engineering from a manufacturing point of view, scaling up clinical grade scaffold production in a cost-effective manner [1]. The goal of an ‘off-the-shelf’ scaffold availability can only be achieved if the entire process is refined, with particular focus on storage conditions. As tissue engineering progresses, tissue preservation increasingly becomes a bottleneck limiting the entire field. The storage, transport and quality control of engineered tissues and organs are vital for success.

Oesophageal tissue engineering is an expanding area where artificial constructs will soon represent a therapeutic alternative to congenital and acquired oesophageal diseases [2–4]. An ideal scaffold for tissue engineering should i) mimic the structure of the tissue/organ that needs to be replaced, ii) facilitate cell delivery and colonisation and iii) be non-immunogenic. With this view in mind, natural-derived decellularised scaffolds appear to represent an optimal bio-engineered option to fit medical needs. Their application in animal models has been studied in replacement of organs such as the intestine [5], kidney [6,7], liver [8,9] and lung [10]. Most importantly, decellularised scaffolds have reached clinical application in humans in the substitution of bladder [11], urethra [12] and trachea [13,14]. Nevertheless, one of the major limitations of decellularised scaffold use is the lack of consensus on the most appropriate storage methodology.

Preservation of extracellular matrix (ECM) composition and micro-architecture is pivotal in facilitating cell-matrix interaction, maintaining an organised 3D structure, retaining important structural and functional signals and stimulating a local immunological response capable of promoting cell survival [5,15–17]. At present, even though several methods have been developed to create decellularised scaffolds, no ideal methodology has been described for long-term storage of decellularised oesophagi [18]. Moreover, despite the advances in scaffold development, the feasibility of the process itself in a large animal model has yet to be developed. In order to advance this work from the laboratory to the clinic it is necessary to identify a storage methodology that allows anticipatory preparation of the scaffold coupled with appropriate bio-banking [19].

In this study we generated a decellularised scaffold from a large animal model and investigated the effect of different storage conditions on ECM component and structure preservation with the aim of identifying a suitable technique for short- and long-term storage.

## Methods

### Organ harvest

Oesophagi were obtained from syngeneic male New Zealand White rabbits (2.0–2.5 kg) following ethics approval by the University College London ethics committee and under UK Home Office Project Licence PPL 70/7504. Rabbit were euthanized by terminal anaesthesia and exsanguination. A midline laparotomy was performed, the oesophagus was harvested up to the gastro-oesophageal junction and washed with phosphate buffered saline containing 1% antibiotic/antimycotic (PBS/AA; Sigma, UK).

### Decellularisation protocol

A detergent-enzymatic treatment (DET) was used for decellularisation as previously described in a porcine model [20]. The oesophageal lumen was perfused with continuous fluid delivery (Masterflex L/S, UK) at 1 ml/min. Each DET cycle was composed of deionised water (dH_2_O) at 4°C for 24 hrs, 4% sodium deoxycholate (SDC; Sigma, UK) at room temperature (RT) for 4 hrs, and 2000 Kunitz DNase-I (Sigma, UK) in 1 M NaCl at RT for 3 hrs. The process was repeated for three cycles.

### Storage protocols

#### Slow cooling medium (SCM)

Samples were cooled slowly (-1°C/min) while immersed in 90% medium (RMPI; Sigma, UK) and 10% dimethyl sulfoxide (Me2SO; Sigma, UK). Slow cooling was achieved using appropriate Nalgene freezing containers kept at -80°C overnight. Samples were then placed and stored in the vapour phase of liquid nitrogen at approximately -160°C. Cooling tanks were screened for temperature fluctuations using an electronic monitoring system. For thawing, oesophagi were rapidly thawed in a 37°C water bath.

#### Four degrees (4°C)

Samples were placed in PBS/AA and stored at 4°C.

### Scaffold analyses

Scaffolds were analysed after each decellularisation cycle and compared to the fresh tissue. Furthermore, scaffold characterisation was performed after 2 weeks, 4 weeks, 3 months and 6 months of storage at the 2 different conditions (SCM and 4°C). A minimum of 4 samples from distinct animals was used for each analysis (n≥4).

#### Histology

Samples were fixed for 24 hours in 10% neutral buffered formalin solution in PBS (pH 7.4; Sigma, UK) at RT, washed in dH_2_O, dehydrated in graded alcohol, embedded in paraffin and sectioned at 5μm. Tissue slides were stained with haematoxylin and eosin (H&E; Leica, Germany), Masson's Trichrome (MT; Leica, Raymond A Lamb, BDH Chemicals Ltd), Elastin Van Gieson (EVG; VWR, Leica, Raymond A Lamb), and Alcian Blue (AB; BDH Chemicals Ltd, Cellpath Ltd) stains.

#### DNA quantification

DNA was isolated using a tissue DNA isolation kit following the manufacturer’s instructions (PureLink Genomic DNA MiniKit, Invitrogen, UK), as previously described [16].

#### ECM component quantification

Collagen, elastin and glycosaminoglycan (GAG) content were quantified as previously described [16] using the total collagen assay kit (Biocolor, UK), the FASTIN elastin assay and the GAG assay kit (Biocolor, UK) respectively.

#### Synchrotron-based x-ray phase contrast imaging (XPCI)

XPCI was performed as previously described [21]. Briefly, measurements were performed at the biomedical beamline (ID17) of the European Synchrotron Radiation Facility in Grenoble, France. The samples were placed approximately 150 m from the source on a PI miCos rotation stage and the detector was placed at 3.45 m from the sample. The beam was monochromatised by a fixed-exit Laue/Laue silicon double crystal to 26 keV (ΔE/E ~ 0.02%) and filtered using 0.8 mm of copper and 3 mm of aluminium. Images were recorded by a FReLoN CCD camera coupled to a 47 μm thick Gd_3_Ga_5_O_12_ scintillator. Images were phase-retrieved using the “single-distance” method developed by Paganin et al, and 3D reconstructions were performed using standard filtered back-projections [22].

#### Chicken chorioallantoic membrane assay (CAM)

The angiogenic properties of the decellularised scaffolds were assessed using the CAM assay, as previously described [23]. Fertilised chicken eggs were incubated at 37°C and constant humidity. At day 3 of incubation an oval window of approximately 3 cm in diameter was cut into the shell and sealed with tape. At day 8 of incubation, 2 mm diameter decellularised scaffolds, negative (polyester soaked in PBS) and positive controls (polyester soaked in 200ng/ml vascular endothelial growth factor; Sigma, UK) were placed on the CAM. Images were taken up day 18 of incubation with a stereomicroscope. The number of blood vessels less than 10 μm in diameter converging towards the placed tissues was counted blindly by multiple assessors (n = 4), with the mean of the counts being considered.

#### Biomechanical testing

To evaluate the biomechanical properties of decellularised oesophagi, specimens were tested and subjected to uniaxial longitudinal tension until failure, as previously described [5]. Uniaxial tension was applied using an Instron 5565, with specimens in the form of flat dumbbells (20 mm) loaded at a constant tension rate of 100 mm/min. The thickness of the samples was measured using a digital electronic micrometer (RS components, US) at three places of the dumbbell and averaged. Four samples were considered for each evaluated time point.

#### Scanning electron microscopy (SEM)

Samples were fixed in 2.5% glutaraldehyde (Sigma, UK) in 0.1 M phosphate buffer and left for 24 hrs at 4°C. SEM was performed as previously described [16].

#### Statistics

Data were calculated and are reported as mean ± standard deviation, unless otherwise stated. Significance for continuous data was determined by performing one-way analysis of variance with post-hoc Bonferroni tests and two-tailed unpaired Student’s t-test. A p-value of less than 0.05 was considered to be significant. Statistical analysis was performed using GraphPad Prism 6 (GraphPad Software, US).

## Results

### Development of a decellularised oesophageal scaffold

**Fig 1:**
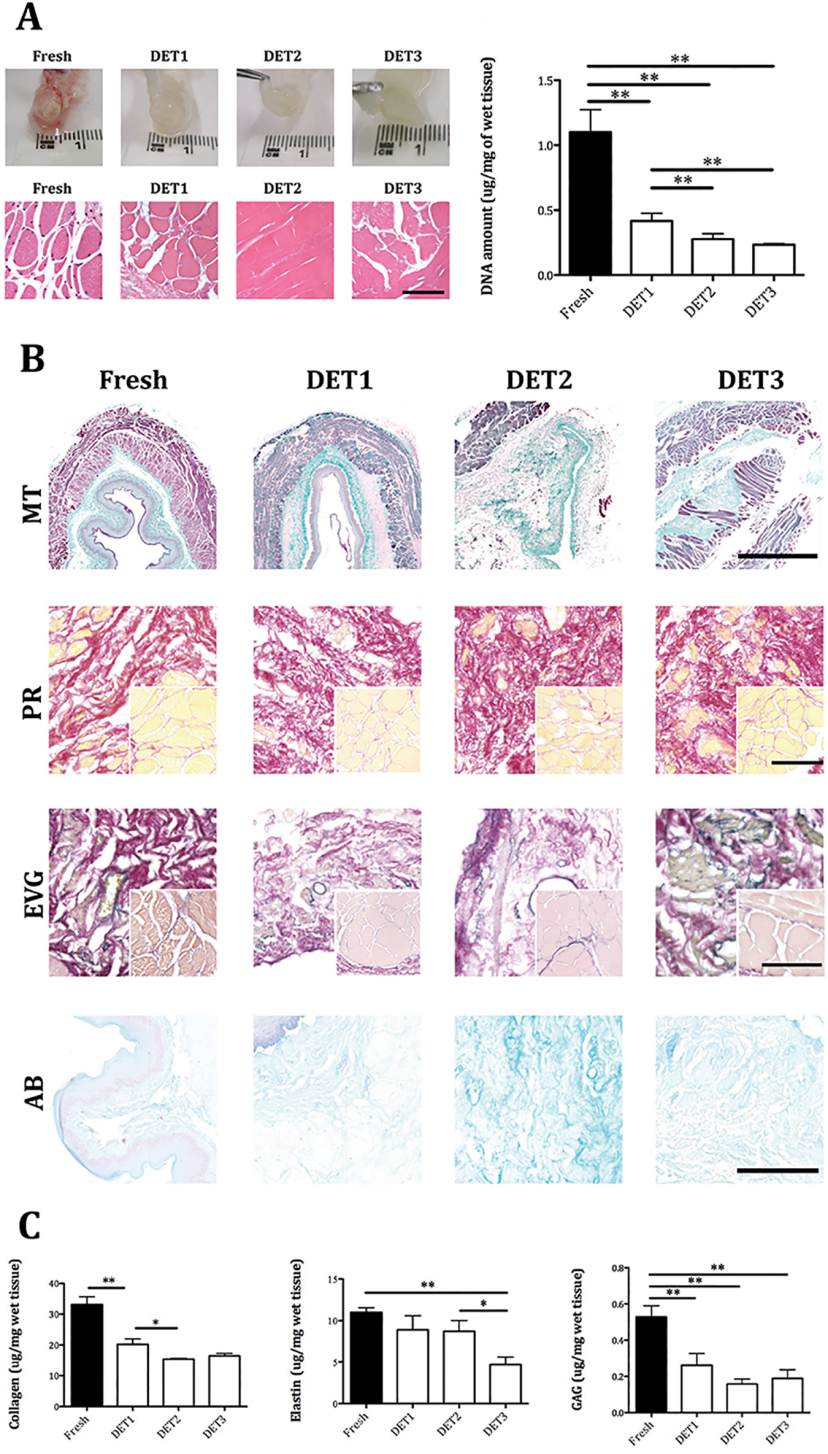
Decellularisation efficiency and scaffolds characterization. (A) Efficient cell removal after 2 DET cycles was evident by macroscopic appearance, H&E staining and DNA quantification. (B) Immunohistochemistry for extracellular matrix composition. Masson’s Trichrome and Picrosirius Red staining demonstrated collagen preservation in both submucosa and among muscle fibers (inset). Elastin Van Gieson staining showed elastic fibers in the submucosa, around blood vessels and surrounding muscle fascicles both in the fresh and DET tissues. Muscular elastin staining was reduced after 3 DET cycles (inset). Alcian Blue staining indicated glycosaminoglycan preservation (bar = 100μm). (C) Extracellular component quantification demonstrated a gradual decrease in collagen after the first and second DET cycle. Elastin decreased after 3 DET cycles. glycosaminoglycan were partially reduced by the first DET cycle. DET = Detergent-Enzymatic Treatment, MT = Masson’s Trichrome, PR = Picrosirius Red, EVG = Elastin Van Gieson, AB = Alcian Blue. *p<0.05; **p<0.01.

Macroscopic imaging showed the oesophagi turning translucent after 1 DET cycle (Fig 1A). H&E staining demonstrated a progressive loss of cells with each cycle of decellularisation (Fig 1A). After 2 DET cycles no nuclei could be detected within the scaffold. Decellularisation efficiency was confirmed by DNA quantification with a significant decrease in the DNA content after each DET cycle compared to the fresh tissue (p<0.01), with no difference between cycles 2 and 3 (Fig 1A). Gel electrophoresis indicated that after DET cycle 2 there was no genomic DNA, since no bands were detected (S1 Fig). The fresh oesophagus sample had a distinct genomic band while at cycle 1 there was a fading band, indicating that the DNA was broken down and gradually removed from the oesophagi samples.

MT staining confirmed nuclear material absence and demonstrated ECM structure preservation in the lamina propria, submucosa and intermuscular septae (Fig 1B, MT). PR staining showed collagen preservation in the submucosa (Fig 1B, PR) and enucleated intermuscular fibres (Fig 1B, PR, inset). EVG staining determined the presence of elastin (black) in the submucosa as circular strands, around blood vessels and surrounding enucleated muscle fascicles. The submucosal and vascular elastin was well preserved (Fig 1B, EVG), with muscular elastin fading following 3 DET cycles (Fig 1B, EVG, inset). AB staining demonstrated GAG preservation (Fig 1B, AB). A significant reduction in collagen content after the first and the second DET cycle was detected (p<0.01 and p<0.05 respectively). While there was no significant loss in elastin after the first 2 DET cycles, 3 DET cycles displayed a significant decrease of elastin amount compared to both the fresh (p<0.01) and 2 DET cycles (p<0.05). Decellularisation also led to lower GAG levels after the first DET cycle (p< 0.01), with no further changes after the following cycles (Fig 1C).

**Fig 2:**
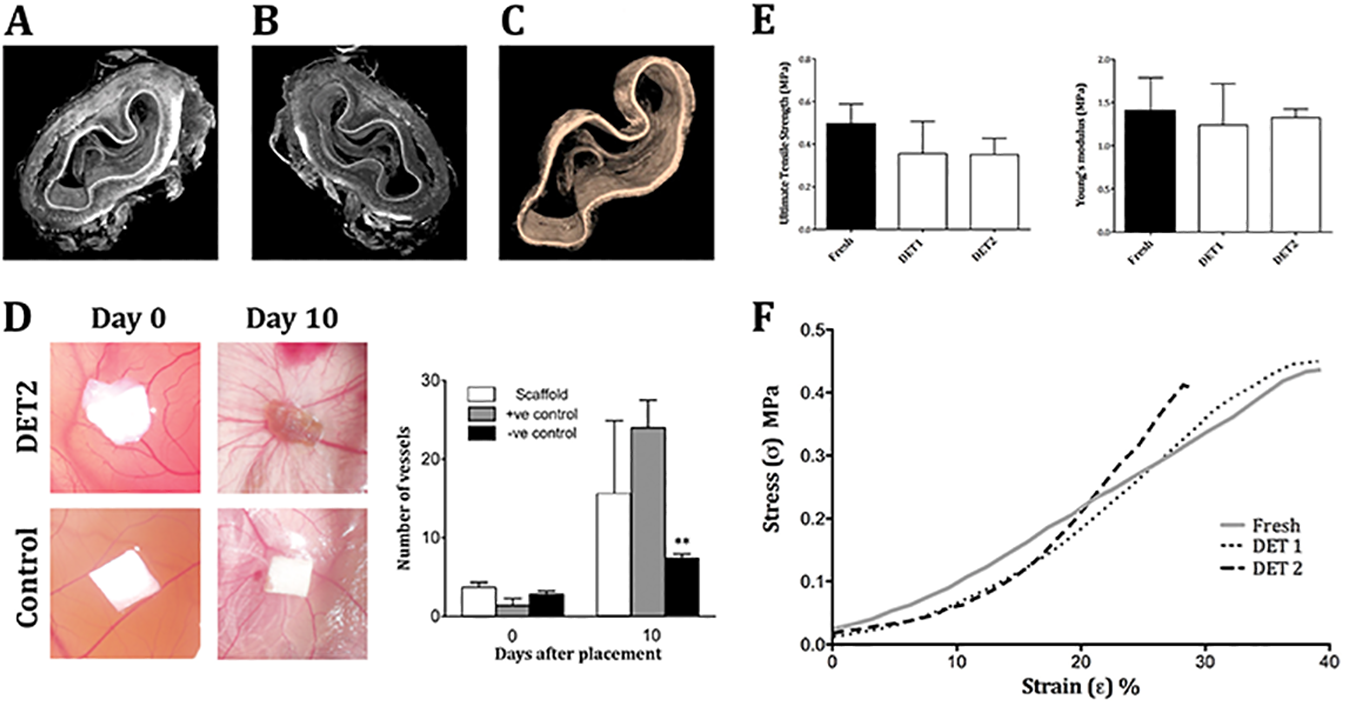
Functional analysis of decellularised scaffolds. (A,B) Synchrotron analysis after 2 DET cycles confirmed extracellular matrix preservation in the lamina propria, submucosa and muscularis. (C) An intact basement membrane was detected across the entire scaffold segment. (D) Blood vessels were seen converging towards the scaffold in a spoked wheel manner after 10 days from placement on the CAM as confirmed by blinded quantification of the vessels compared to the negative control (**p< 0.01). (E) The maximum tensile stress at which the samples broke and the elasticity modulus remained comparable to fresh after decellularisation. (F) Characteristic stress-strain curves showing that by increasing the number of DET cycles the tensile stress at which the samples break remains the same as seen in E. DET = Detergent-Enzymatic Treatment, CAM = Chicken chorioallantoic membrane assay.

Synchrotron-based XPCI imaging allowed a deeper investigation of preservation of the micro-architecture across a large scaffold segment. The analysis of the decellularised scaffold after 2 DET cycles confirmed ECM preservation in both the lamina propria and submucosa (Fig 2A). The muscularis mucosae showed maintained morphology and organisation (Fig 2B). Furthermore, an intact basement membrane was also evident in the cross section image (Fig 2C). 3D reconstruction demonstrated preservation of the muscularis and the basement membrane across the whole thickness of the tissue (S1 Video).

Following placement on the CAM numerous blood vessels were seen converging towards the scaffold in a spoke wheel manner while fewer vessels were detected in the negative control sample (Fig 2D). Blinded quantification of the converging blood vessels indicated that there was a significant difference in the number of vessels attracted by the scaffold at day 10 compared to day 0 after seeding (p<0.01) and to the negative control at day 10 (p<0.01). There were no significant differences between the scaffold and the positive control loaded with VEGF (Fig 2D).

Decellularised scaffolds were analysed for stiffness and elasticity using the stress/strain curve, ultimate tensile stress (UTS) and Young's Modulus. There was no significant difference in the UTS among fresh and decellularised samples after 1 or 2 cycles (Fig 2E). Characteristic stress-strain curves showed that with increasing number of DET cycles the curve became steeper and more S-shaped, while the tensile stress at which the samples break remained the same (Fig 2F).

### Comparison of storage protocols

**Fig 3:**
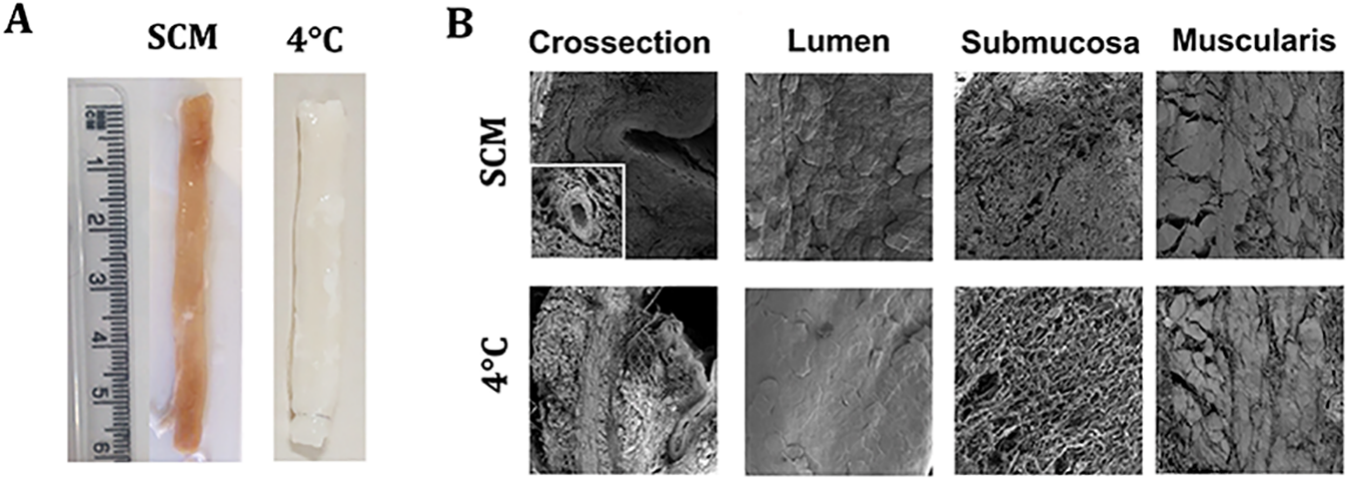
Macro- and microscopic appearance of stored decellularised scaffolds at 6 months. (A) Macroscopic appearances varied in the two protocols. (B) Scanning electron microscopy analysis. While SCM scaffolds demonstrated a good preservation of all oesophageal layers, in 4°C samples extracellular matrix was falling apart with signs of degradation. SCM = slow cooling medium, 4°C = 4°C in PBS.

From a macroscopic perspective, SCM scaffolds had a pink hue due to immersion in medium supplemented with phenol red. Except for the colour, SCM samples were comparable with freshly decellularised scaffolds. While 4°C samples stored for 2 and 4 weeks were macroscopically comparable to freshly decellularised oesophagi, after storing for longer periods the matrix showed degradation and loss of consistency (Fig 3A).

**Fig 4:**
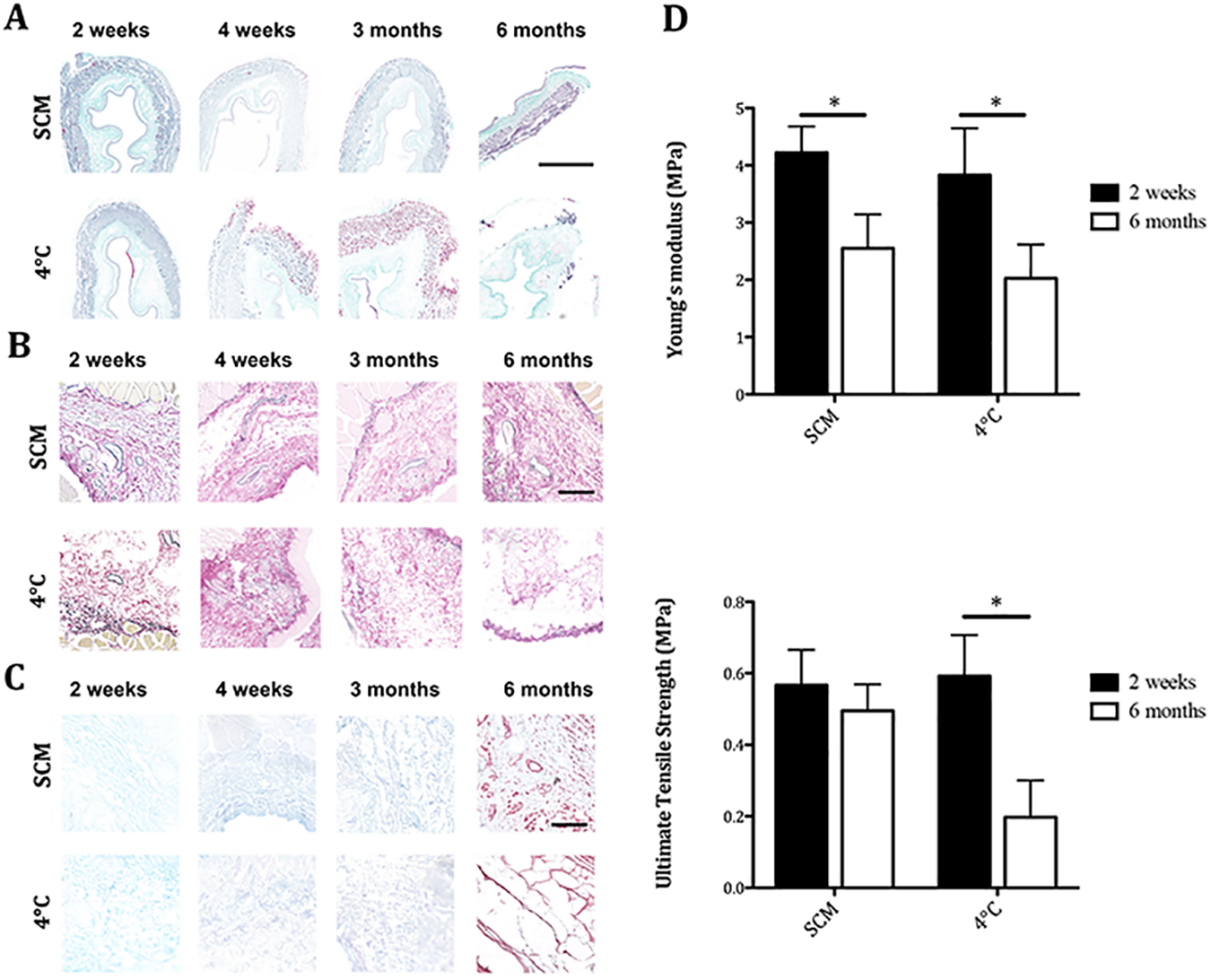
Composition and mechanical properties of decellularised scaffolds after storage. (A) Masson’s Trichrome staining demonstrated a progressive loss of architecture in 4°C-treated scaffolds. (B) Elastin staining showed maintenance of this protein in SCM scaffolds. Elastin was progressively lost in 4°C scaffolds. (C) Alcian Blue staining showed glycosaminoglycan maintenance in both storage methods. (D) Samples stored for 6 months with SCM maintained comparable ultimate tensile stress with 2 week-stored scaffolds despite a decreased Young's modulus with no impact on the maximum stress that the material could withstand. While 4°C samples at 2 weeks showed similar values to SCM, prolonged 4°C storage had a profound impact on the scaffold with a reduction of both values. *p<0.05 (bar = 100μm). SCM = slow cooling medium, 4°C = 4°C in PBS.

SEM was performed for further understanding of scaffold ultrastructure. In Fig 4B we report the images obtained for the 2 storage methods at the last time point (6 months), with a subdivision for the different structural elements: lumen, submucosa and muscularis. Cross-sections of SCM oesophagi showed a similar preservation of the strata with detectable intact blood vessels in the submucosa of the SCM scaffold (insert). The 4°C-stored oesophagi demonstrated detachment of the mucosa and layers of the muscularis. The ECM was destroyed with collagen and elastin fibres forming spherical bundles. Lumen examination at higher magnification showed no substantial differences between protocols, with preservation of the ridges of the (now acellular) stratified epithelium. Analysis of submucosa showed clear differences between storage methodologies: SCM samples had a compact submucosa with the collagen fibres still present in bundles, whereas the submucosa in the 4°C samples was completely disorganised with the collagen bundles dispersed as single fibres. The muscularis was well preserved in SCM oesophagi with clear demarcation between the inner circular and outer longitudinal layers, while it was only partially preserved with fragmented bundles in 4°C samples (Fig 3B).

Histological analysis showed further differences between the storage methods. MT staining demonstrated preserved ECM architecture in SCM-stored oesophagi throughout all time points. 4°C-stored scaffolds showed good preservation of the collagen up to 4 weeks, with progressive deterioration in later time points, namely increasingly thinning muscle bundles and detachment (Fig 4A). EVG staining highlighted changes in the elastin fibres present in the ECM. SCM-stored oesophagi demonstrated a well-maintained presence of vascular and submucosal elastin. Conservation of the perimuscular elastin was similar across samples and time points. 4°C-stored samples demonstrated a good preservation of vascular and submucosal elastin only at the first time point, after which the submucosa was increasingly disorganised with poor and scattered elastin staining (Fig 4B). AB staining in the stored oesophagi showed preservation of GAG in the submucosa across protocols and time points (Fig 4C).

A deeper analysis was performed at each time point using quantitative assays. No significant differences in GAG, collagen or elastin were detected between the SCM or 4°C storage time points (Table 1).

**Table 1:** Quantification of extracellular matrix components in samples stored with the SCM protocol.

	DET2	2 weeks in SCM	4 weeks in SCM	3 months in SCM	6 months in SCM
**COLLAGEN ug/mg**	15.40 ± 0.28	19.09 ± 3.32	16.14 ± 2.04	25.62 ± 2.86**	27.27 ± 7.87**
**ELASTIN ug/mg**	8.71 ± 2.92	8.64 ± 3.66	12.94 ± 4.54	11.27 ± 0.65	11.92 ± 2.01
**GAG ug/mg**	0.16 ± 0.05	0.24 ± 0.06*	0.25 ± 0.06*	0.29 ± 0.06*	0.30 ± 0.03*

Extracellular matrix content in decellularised rabbit oesophagi prior and after SCM storage. A relative increase of collagen and glycosaminoglycan (GAG) content was detected in stored samples. No significant differences were found between 2 weeks storage and the following time points for each of the analysed components.

*p<0.05

**p<0.01.

Furthermore, biomechanical testing was used to evaluate four samples for each condition. Comparison of Young's modulus and UTS at the first and last time points (2 weeks and 6 months) showed that samples stored in SCM maintained the same UTS after long-term storage despite a decreased Young's modulus. Conversely, while 4°C-stored samples showed UTS and Young's modulus values similar to the SCM group at 2 weeks there were significant differences following 6 months of storage. Prolonged 4°C storage had a profound impact on the scaffold with a reduction in both values. The material became less resistant and could not withstand the applied stress, with lower UTS compared both to the previous time point and to SCM group (Fig 4D).

## Discussion

The rapid evolution of the tissue engineering field is pushing decellularised scaffolds into pre-clinical and clinical use, highlighting limitations that have not been fully addressed yet. In particular, the lack of an established methodology for accurate long-term scaffold storage could limit clinical application. For consistent and controlled use, scaffolds need to be efficiently stored and made readily available for surgical implantation. Therefore, we studied the short- and long-term storage feasibility of a decellularised oesophageal scaffold from a large animal model that could potentially be used for tissue engineering approaches. After having identified an effective decellularisation protocol and used it to prepare clinical grade scaffolds [24], we compared the use of a cryoprotectant with standard storage of acellular scaffolds to verify the potential of this storage method. We show here for the first time that the decellularised oesophagus could be preserved long-term using Me2SO and liquid nitrogen, a well-established methodology for cell storage.

Firstly, we have proven that the DET protocol could be applied for rabbit oesophagus decellularisation, similarly to what we have shown for other organs and species [5–9,14,20,23–25]. DNA quantification, staining and gel electrophoresis showed effective DNA removal after 2 DET cycles with no differences in DNA content found after further cycles. Structural analyses were performed in order to verify that the ECM architecture was appropriately preserved. While multiple staining assays (MT and AB) showed a good preservation of collagen fibres and GAG after decellularisation, staining for elastin (EVG) proved a clear decline of elastin presence after the 3 DET cycles. Quantification of matrix composition confirmed no differences in collagen and GAG content between DET cycle 2 and 3, but a clear drop in elastin content. Synchrotron images confirmed the preservation of the different strata. Based on these findings we proved that 2 DET cycles were efficient in removing the cellular components while preserving ECM integrity. The drop in ECM components at cycle 3 demonstrates that excessive decellularisation destroys the matrix, as shown previously [16]. The presence of enucleated muscle fibres after decellularisation has been reported in other studies where the DET protocol was used for the decellularisation of muscles [20,26]. This result is not related to low decellularisation efficiency and seems to be important for the mechanical properties of the resulting matrix. Assessment of the mechanical properties showed that the decellularised scaffold did not lose its elastic characteristics and its ability to withstand a maximum stress when a longitudinal force was applied to its extremities, corresponding to a preserved Young’s modulus and UTS respectively. Moreover, the scaffold elicited a pro-angiogenic effect as seen at the CAM assay, which would facilitate its integration within the host tissue. These results are in line with our previous studies on DET application for tissue decellularisation in the trachea, lung, liver and small intestine [5,16,24,25,27]. The same protocol has been previously successfully applied for the decellularisation of porcine oesophagi, allowing the development of a scaffold which preserved biomechanical characteristics of the original tissue [20]. In this study we confirm the versatility and reliability of the DET treatment in achieving a good decellularisation in another large animal model.

Following scaffold development, we compared two methodologies commonly used for tissue storage. Cryobiology was first developed in 1949 when Polge discovered the cryoprotective properties of glycerol when storing cells at low temperatures [28]. Since then, other stabilising agents have been used including Me2SO, a cell-permeating agent able to protect proteins from denaturation through electrostatic interactions and reduce the rates of ice nucleation and crystal growth [29]. The current gold standard for preserving cells and allografts is controlled rate freezing with Me2SO. Slow cooling coupled with Me2SO cryoprotection has been shown to preserve ECM integrity following heart valve storage [30]. We thus used slow cooling (-1°C/min) of tissues immersed in 90% medium and 10% Me2SO followed by storage in liquid nitrogen vapour as a first comparison (i.e. SCM).

Storage at 4°C in PBS/AA is another common storage protocol, especially for short-term periods. It has been previously proven that the immunological and mechanical characteristics of decellularised pig scaffolds were unaffected by a 2-month storage in PBS at 4°C [25]. Analyses at later time points with storage of decellularised tracheas stored at 4°C for 1 year showed generalised damage of the ECM architecture and mechanical properties, being considered unsuitable for cell seeding and transplantation [31]. Bonenfant et al evaluated 4°C storage for decellularised mouse lungs demonstrating ECM damage after 3 months [32]. Thus, the second group we compared was storage in PBS/AA at 4°C.

We analysed the scaffolds after short- and long-term storage to mimic the clinical need of off-the-shelf availability. From a macroscopic perspective, no major differences were noted in the short-term, apart from a pink nuance of the SCM scaffold due to the medium used, that could be solved by avoiding phenol red in future applications. On the contrary, long-term storage led to major changes in 4°C samples with clear loss of consistency. From an ultrastructural perspective, SEM analysis confirmed the superiority of the SCM storage protocol in long-term preservation of the architecture and the characteristics of the scaffold’s strata: lumen, submucosa and muscularis.

Studying the structure of the stored scaffolds with histology and quantification assays for ECM components, we found that collagen fibres were preserved in SCM and 4°C samples after 2 and 4 weeks of storage, but analysis performed after 3 and 6 months showed collagen preservation only in SCM samples. These results, confirmed the notion that the 4°C methodology is appropriate only for short-term storage. No major changes were detectable in GAG disposition between protocols, while elastin fibres were clearly well preserved at 3 and 6 months only in SCM samples. To confirm the ability of the SCM protocol to preserve ECM composition both after short and long-term time points, we quantified these components detecting no changes or an increase in collagen/elastin/GAG composition during SCM storage period, from 2 weeks up to 6 months. This increase could be due to the fact that we perform the normalization of collagen/GAG amount on the weight of wet tissue, which is not ideal. The fresh tissue contains cells and other molecules that are replaced by water after decellularization, making very difficult to compare weights before/after decellularization. The ideal experimental set up would be to measure the weight of a same tissue sample fresh and after decellularization for normalization of amount of collagen/GAG on the total weight. This is extremely difficult to perform as the decellularization is conducted on the whole oesophagus and not on single small tissue segments as for the collagen assay. Another way to avoid the total reference weight mismatch would be using dry tissue (for both fresh and decellularized), but this would introduce further variables related to time and technique of re-hydration. Another hypothesis is that after SCM, a certain amount of insoluble cross-linked collagen from the ECM becomes available to the extraction of hydroxyproline, affecting the total quantification which was carried out by hydroxyproline determination. This was also affecting the biomechanical properties of the scaffold after SCM storage, as demonstrated by the tests performed to assess whether the structural preservation allowed a functional advantage. Despite a decrease of the Young’s Modulus after 6 months compared to 2 weeks post storage, the UTS did not differ over time showing that while scaffolds became less stiff they did not lose the ability to respond to a longitudinal stress and withstand the applied force. This was opposed to what detected in 4°C-stored samples, showing a decrease in Young's modulus with a concomitant reduction in UTS proving that the scaffold lost both elasticity and strength.

All together these data demonstrate the superiority of the SCM protocol for long-term storage, suggesting it would be an appropriate choice for use to reach the off-the shelf availability required by clinical translation. While this technique has been widely tested for cell freezing and banking, its efficiency in decellularised oesophageal scaffolds storage was still not formally analysed and confirmed. A number of studies have extrapolated the cell storage technique based on Me2SO/medium immersion and slow cooling in other tissues. Several studies on the cryopreservation of heart valves have shown that conventional cryopreservation causes alteration of crucial leaflet matrix structures [30,33,34]. Interestingly, biochemical testing demonstrated no significant differences between the amounts of collagen, desmosine, and elastin of fresh and cryopreserved specimens [33]. In one of these papers, slow cooling of porcine heart valves with Me2SO was also compared to ice-free cryopreservation using a vitrification solution. The two approaches showed overall comparable results, with lower, but not statistically significant, ECM preservation with the first technique, while leaflets and artery tissues were significantly less viable in the latter. Nevertheless, these studies were conducted on fresh porcine heart valves without any decellularization process. The absence of cells in a cryopreserved ECM-derived scaffold could change the effect of the storage method on the structure, composition and biomechanical properties of the tissue of interest. Gallo et al decellularised porcine aortic valve conduits and randomised 12 Vietnamese Pigs to receive either decellularised or decellularised and cryopreserved conduits in a transplantation model [35]. No signs of dilatation, stenosis or regurgitation were seen in either group by echocardiography and cryopreserved conduits allowed cell repopulation and tissue renewal, although demonstrating signs of deterioration in some areas, suggesting a need to fine-tune the cryopreservation protocol. A comparison with specific ice-free cryopreservation protocols (vitrification) for rabbit decellularized oesophagi would be interesting [36], providing further insight for the determination of the best storage technique without affecting the important feature of the ECM.

It is known that different decellularisation methods can affect composition, structure and biomechanical properties of the resulting ECM [37]. Nevertheless, even though we compared the efficacy of the 2 storage techniques only on DET-derived decellularised scaffolds, we believe that the SCM protocol, due to the mechanisms of cryoprotection, will allow optimal preservation of other decellularised scaffolds regardless of species origin, size or decellularisation methodology. A major challenge will be to optimise an effective homogeneous delivery of the cryoprotectant when bigger or more complex organs will be processed for storage.

## Conclusions

Tissue engineering is becoming a valid alternative for organ replacement owing to the improvements in natural scaffold production. However, moving from bench-to-bedside requires the development of new strategies for scaffold storage and shipping. In this study we successfully decellularised rabbit oesophagi using the previously described DET method. Most importantly, we demonstrated how slow cooling in medium and Me2SO following by long-term storage in liquid nitrogen vapour represents an effective technique for preservation of the structural and mechanical properties of the decellularised scaffold.

## Supporting information

S1 Fig Gel electrophoresis. Gel electrophoresis indicated the lack of genomic DNA after DET cycle 2.
(TIF)

S1 Video Synchrotron-based XPCI. The scaffold after 3 DET cycles confirmed ECM preservation in the lamina propria, submucosa and muscularis. An intact basement membrane was detected across the whole scaffold segment.
(MOV)
