# Supplementary figures and images for: Long-term cryopreservation of decellularised oesophagi for tissue engineering clinical application
Source: PLoS One. 2017 Jun 9;12(6):e0179341. doi: 10.1371/journal.pone.0179341 (PMC5466304; doi:10.1371/journal.pone.0179341)

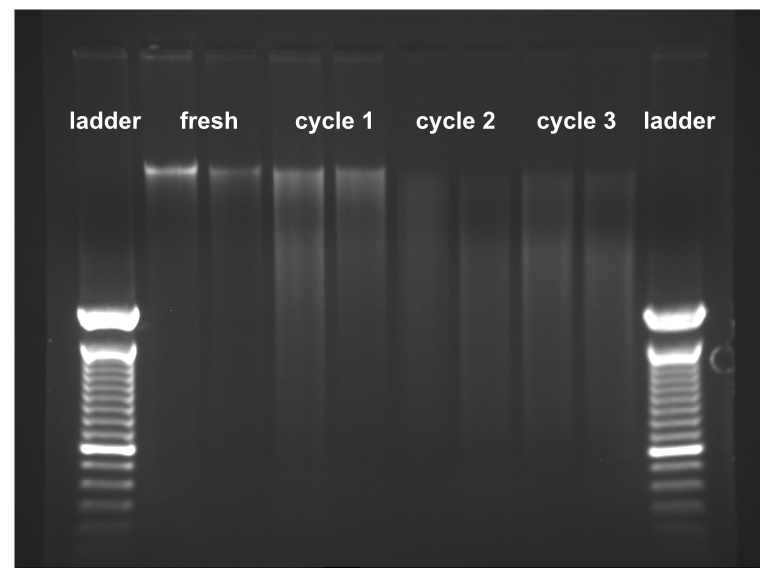

Supplement: S1 Fig — Gel electrophoresis indicated the lack of genomic DNA after DET cycle 2. (TIF) [file pone.0179341.s001.tif]
